# Curating the Health Humanities: Perspectives from Literary Studies

**DOI:** 10.1007/s10912-025-09968-z

**Published:** 2025-08-13

**Authors:** Jade French, Sara Read

**Affiliations:** Loughborough University, Epinal Way, Loughborough, LE11 3TU UK

**Keywords:** Health humanities, Literary studies, Narrative, Material culture, Visual art

## Abstract

This reflective review examines the curatorial possibilities of bringing literary scholars, archivists, makers, and artists into dialogue through an exhibition organized by the Health Humanities Research Group at Loughborough University in 2023. We reflect on how material culture, visual art, historical objects, and archives are part of our practice as literary scholars and the collaborative potential this engenders. The objects on display ranged from historical pieces, such as an early modern birthing stool, to contemporary creative works, including textiles, found poems, and digital collages. Placing these different elements side by side allowed us to think about how material culture, literary criticism, and artistic practice can speak to one another. Together, the exhibition aimed to challenge any simplistic division between health and illness, instead drawing attention to the personal and shared stories that shape our experiences of the various stages of our lives.

What does it mean to curate the health humanities as literary scholars? In 2023, health humanities researchers, based in the Department of English at Loughborough University, sought to explore this question. Our approach corresponds with how Lizzie Muller and Caroline Seck Langhill “describe… objects as *lively* to convey the feeling that they speak, offer suggestions, make demands and pose problems” (2022, 1, italics in original). For the exhibition, the objects, archives, and artworks curated were chosen precisely because they were challenging, intriguing, funny, and sometimes troubling, but all demanded a response from the viewer. In this review, we reflect on how our research as literary scholars relates to the exhibition, whether that’s remediating the books and archives that are the tools of our trade, mobilizing different forms of narrative, and creating installations for creative writing.

The starting point for this exhibition came from a conversation between [Sara Read and Claire O’Callaghan] about both owning objects that related to their research. For [Read], that was a model of an early-modern birthing stool and a nineteenth-century antique one, and for [O’Callaghan], a replica of the sofa which Emily Brontë reputedly died on and which has become known as the death couch (Fig. 1). The artifact had been commissioned for the 2022 film *Emily* (directed by Frances O’Connor) and was gifted to [O’Callaghan]. This conversation then broadened out to the whole Health Humanities Research Group, where we considered how these objects—along with others in the collections of other members—offered a way to think about life cycles and temporality. The exhibition was curated by [Jade French and Sara Read], in collaboration with the group, whose research areas span across historical periods.^1^

**Fig. 1:**
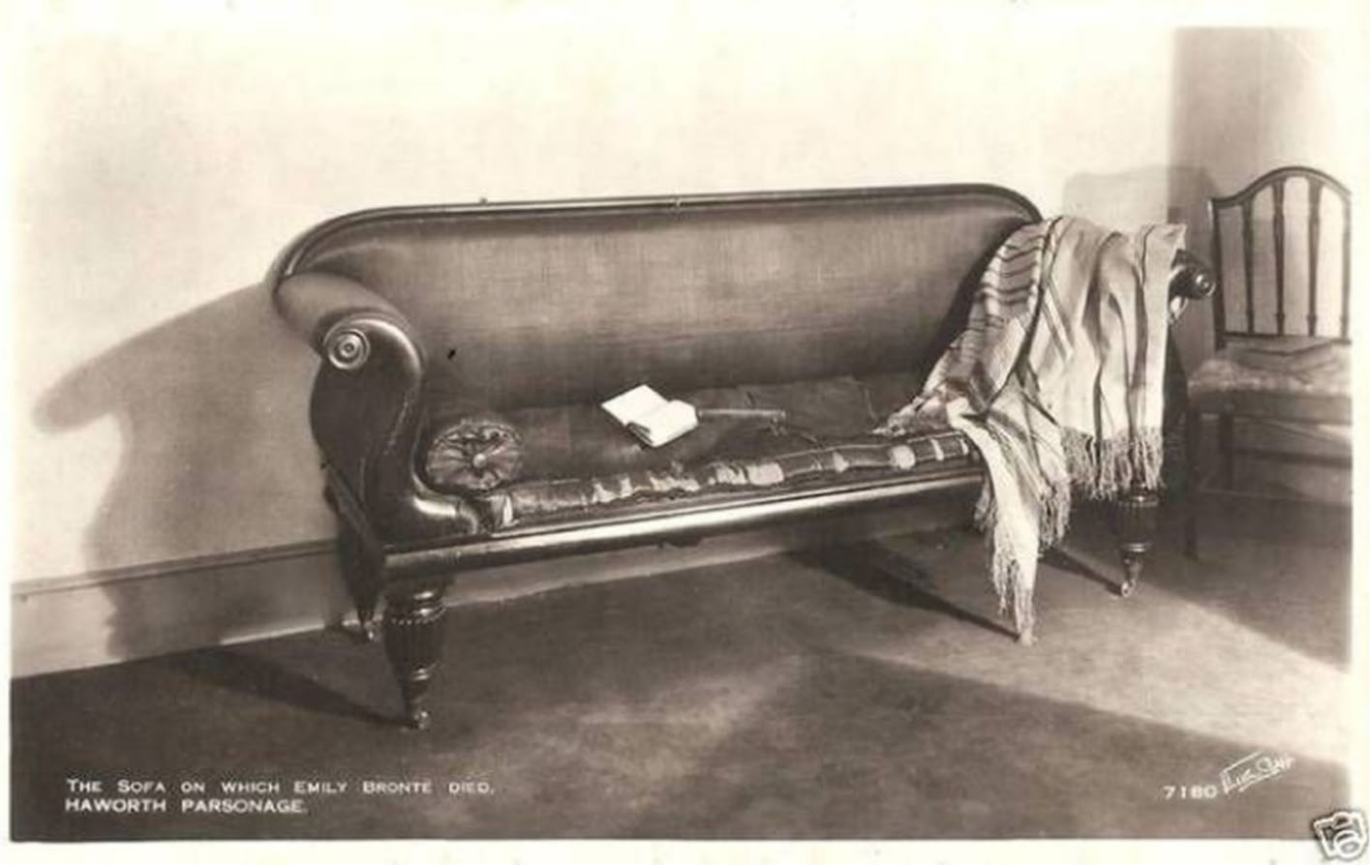
Postcard of sofa, a replica of which is now owned by Dr Claire O’Callaghan

Curating exhibitions in the health humanities involves navigating ethical, intellectual, and practical challenges to effectively engage diverse audiences. A workshop at Birkbeck, University of London in 2018, run by Fiona Johnstone and Heather Tilley, and titled “Curating the Medical Humanities,” addressed “knotty questions” (Barratt 2018), emphasizing considerations of audience, accessibility, participation, and public engagement, which informed our practice too. Following this, some questions we kept in mind were as follows: What does it mean to tell stories about health and illness? Is the binary between health and ill health ever so straightforward? How can objects disrupt this binary? The researchers and artists who came together for this exhibition explored these questions in a variety of creative ways: poetry and essays met textiles, archival objects, and artworks all responding to the impact of health in society and culture across time.

Bringing all these different forms together meant thinking about a variety of different practices, from representing creative writing to narrative materials produced in public workshops. While the Health Humanities Research Group is based in the Department of English, members solicited objects from humanities scholars across campus. We were then tasked with curating various strands of people’s research across different time periods, different materials, and different objects. We decided to try and draw out thematic links rather than chronological ones. The first major theme that emerged was that of “life spans,” with objects representing birth, adolescence, midlife, and old age, represented in our subtitle: from birthing stools to death couches. A second theme highlighted community and personal narratives, and a third examined the “social model of disability” (Shakespeare 2010), which shows howargue disability is caused by the way society is organized rather than by a person’s impairment or difference. Through these themes, we were able to draw links across the exhibition and focus on different forms of narrative.

We included creative pieces that directly worked with communities and reflected on personal experience. Tamarin Norwood’s “From The Heart Notelets” (2023–) features a new resource for families who have lost a baby (Fig. 2). Norwood made packs of 25 note cards created in collaboration with the baby loss charity Held in Our Hearts, with sensitive visual design by Josh Nuttall. The cards were informed by Norwood’s own maternal experience of baby loss and her research into the potential of creative forms of expression in bereavement. We also collaborated with Augusta Philippou at the symposium and invited her to show her work, “Connection: Emotion: Memory.” These large-scale textiles offer a visual representation of the transience of memory (Fig. 3). We also displayed textile squares produced during a series of “Dementia Cafes” run with the Alzheimer’s Society. Each square was created to form a large-scale patchwork to visually represent the sense of community that was present in the Dementia Cafes. Drawing on personal experience, Swati Joshi’s “Documents of my Breath” (2022) charts her experiences in childhood with chronic bronchitis and breathlessness. When she finally found a treatment that worked for her, she began creating colorful artworks by blowing ink through a homemade bubble-maker. In these works, she describes her journey from patient to artist. By documenting the dialogue between her breath, cough, and phlegm, Joshi offers an experimental life writing. Through different narrative approaches, each of these contributions offered affective experiences through different mediums.

**Fig. 2:**
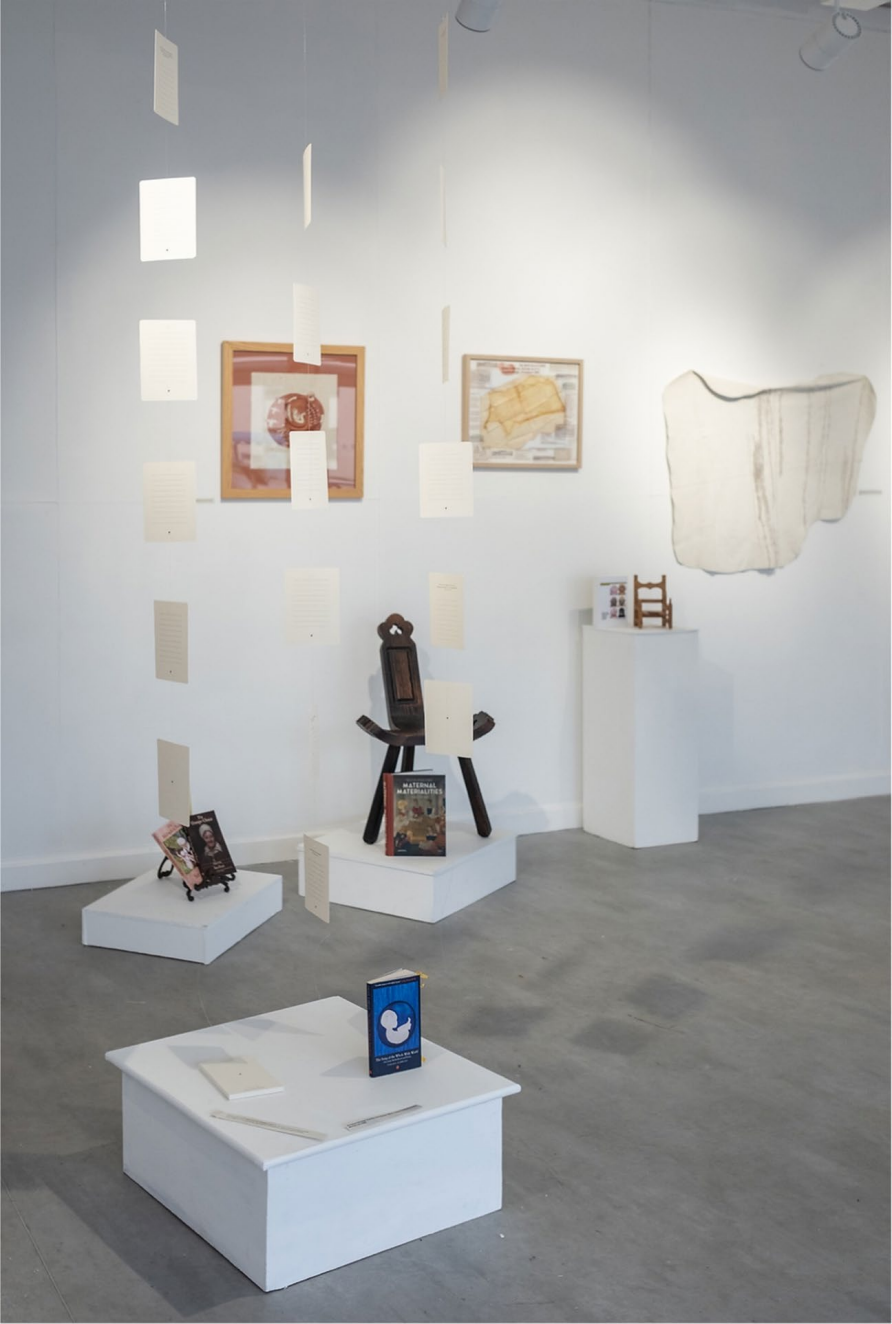
Installation shot, Martin Hall Exhibition Space. Featuring Dr Tamarin Norwood’s “From the Heart Notelets” (2023–), Dr Sara Read’s early-modern installation on birth, and Augusta Philippou’s textile. Image credit LU Arts, Loughborough University

**Fig. 3:**
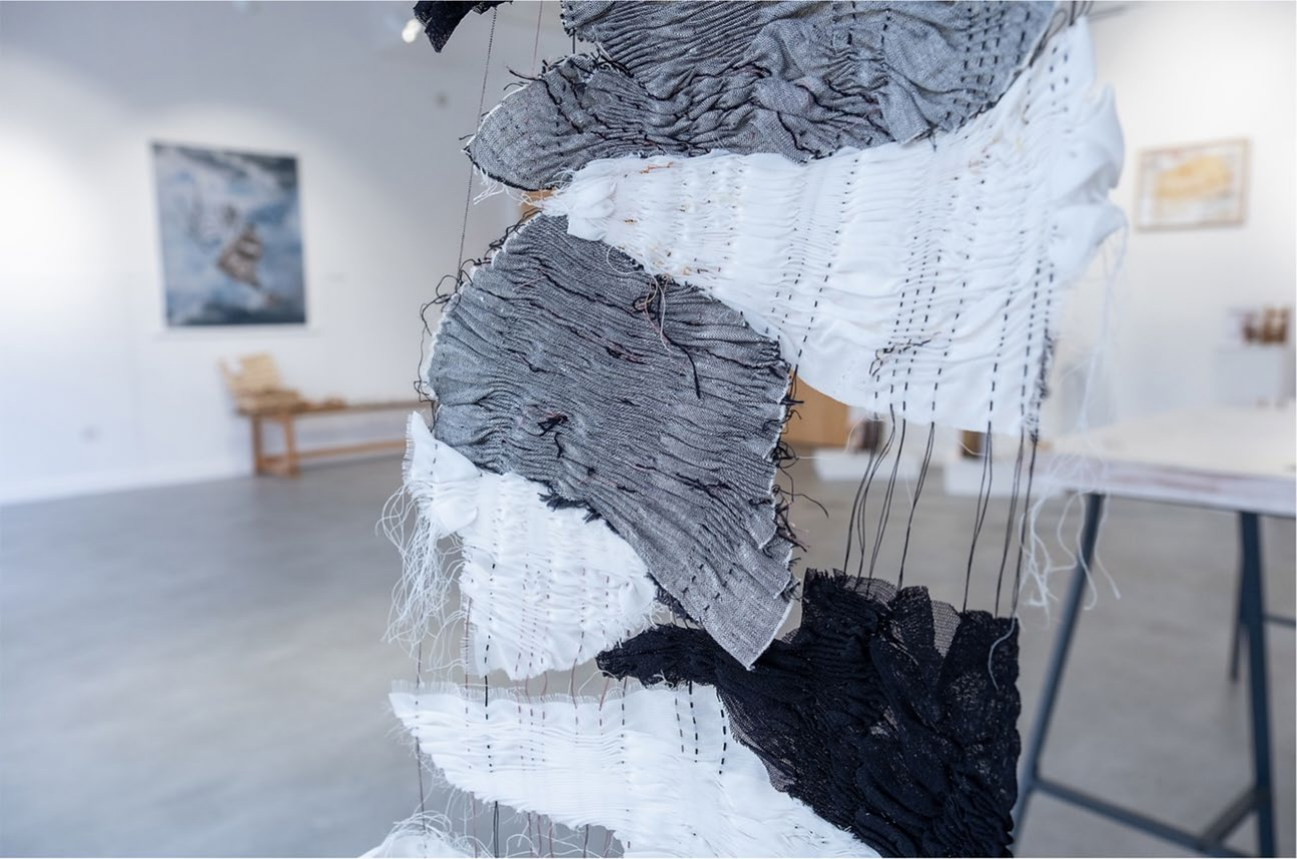
Augusta Philippou’s detail from textile piece as part of “Connection: Emotion: Memory.” Image credit LU Arts, Loughborough University

Artists and researchers also turned themes from novels about health, illness, and wellbeing into new forms. Printmaker Becca Thorne presented “The Gossips’ Choice” (2021), a linoprint inspired by Sara Read’s novel of the same name, suggesting research-led art forms such as historical novels can inspire further art. Thorne’s design drew on symbols of early modern medicine, highlighting the role of care central to Read’s novel. Ryan French’s digital collage “Croning the Canon” (2024) explored post-human elements of aging in Leonora Carrington’s *The Hearing Trumpet* (1974). French likens the creative process to that of alchemy and in this piece turned the novel’s themes of aging into a liberatory image of transformation (Fig. 4). Doctoral researcher, Hannah Palmer’s embroidery “Cara” depicted a scene from Ménie Muriel Dowie’s *Gallia* (1895). Dowie’s novel is analyzed in Hannah’s doctoral thesis “Abortion in Nineteenth-Century British Literature,” which examines various approaches to terminating pregnancy in nineteenth-century literature, life-writing, and culture. The act of stitching acts as a mode of close reading, turning a moment from the novel into a tactile object that invites a feminist and material lens of both. Fictional texts became generative, creative sources that were remediated into new forms. The works were not merely illustrating the novels; rather, they conceptually engaged with different research focuses (Fig. 5).

**Fig. 4:**
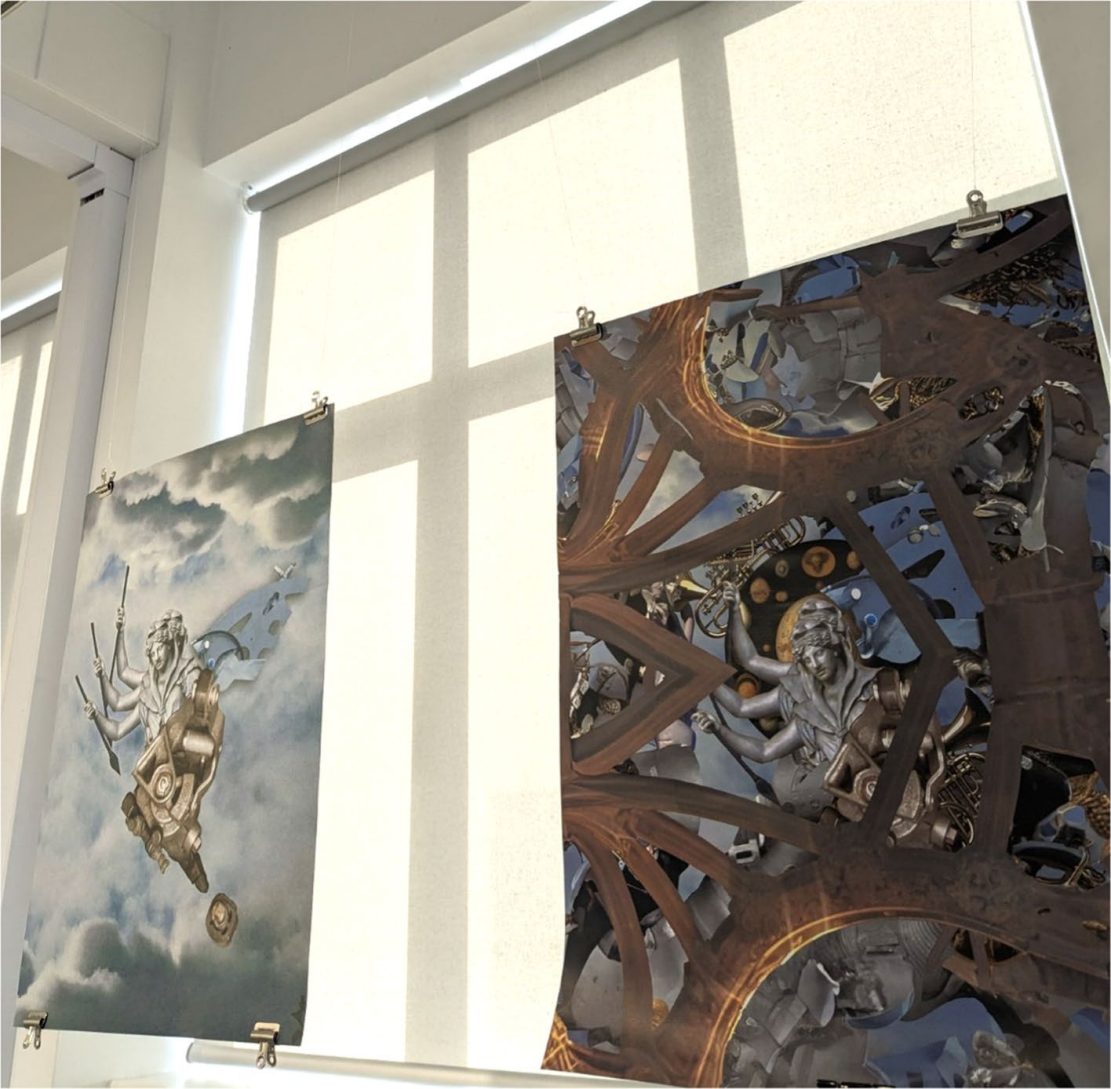
Ryan Peter French’s “Croning the Canon,” 2024. Image credit LU Arts, Loughborough University

**Fig. 5:**
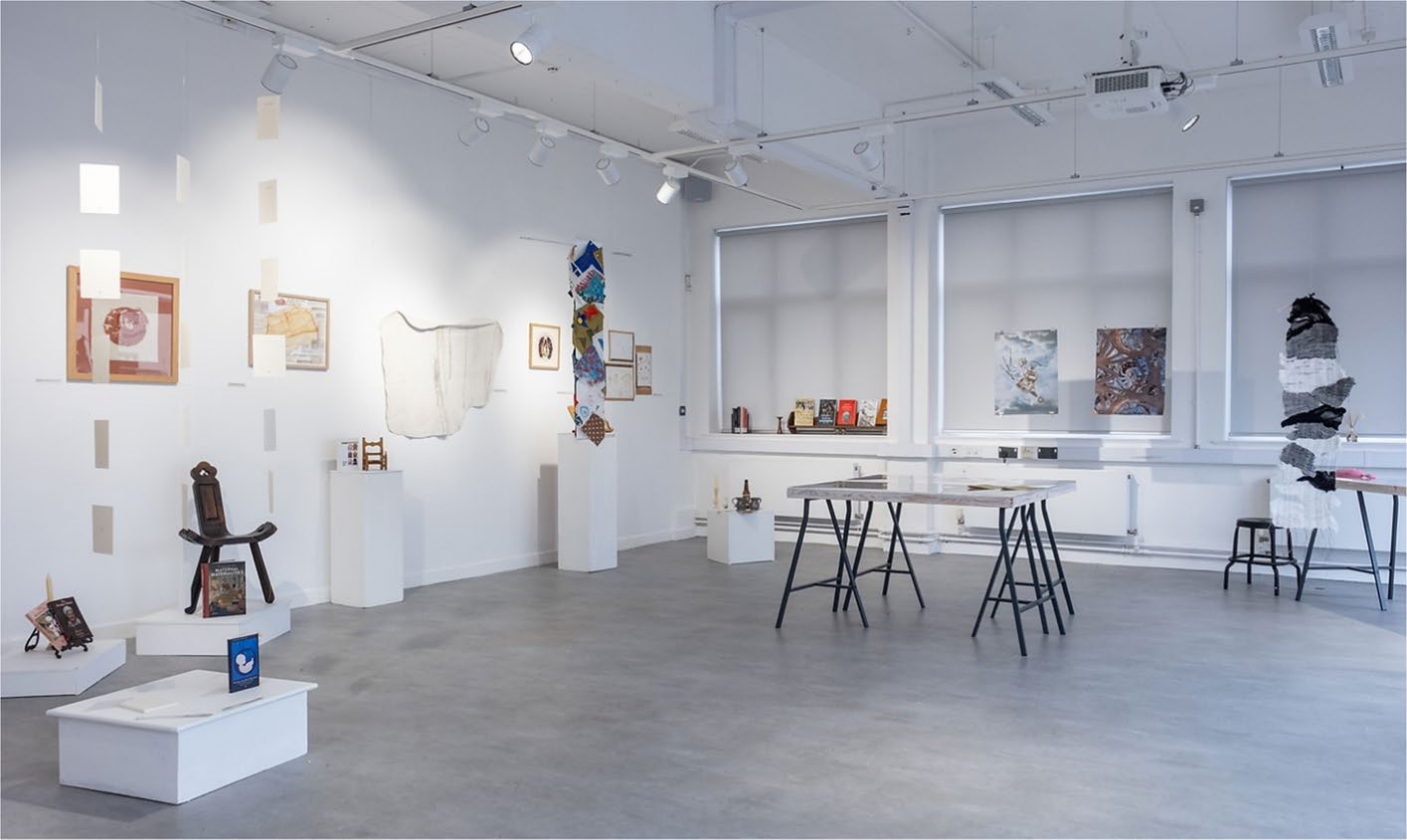
Installation shot, Martin Hall Exhibition Space, Loughborough. Image credit: LU Arts, Loughborough University

The importance of historical artifacts and archives was also a concern of the exhibition. We wanted to display archival material as a conceptual and material presence that resists or invites interpretation. Sara Read displayed the historical objects she has collected, including an artwork framed by newspaper advertisements for cauls—inspired by a birth caul donated by a local Loughborough woman from her mother’s birth in 1904—which fetched good money in Victorian England as they were associated with good luck. Read also commissioned woodworker, James Cogbill, to create a 20-cm model of the birth chair used by midwife Lucie Smith in Sara’s historical novels. These historical artifacts underpin Read’s creative process. To represent the dynamic textual resources of our researchers’ work, we also used vitrines to exhibit examples of archival research and poetry by various researchers. Anne-Marie Beller, Kerry Featherstone, and Claire O’Callaghan have worked extensively with the Somerset Pauper Lunatic Asylum archive, held at the Somerset Heritage Centre at Taunton. The rich archive holds detailed records and offers valuable insight into daily life in the asylum in the nineteenth century, and this research is feeding into several projects-in-progress. We displayed the found poem, made from fragments of texts found in the archive, alongside further research to contextualize the creative response. We displayed Dr Jennifer Cooke’s poem, “Song O’Lock,” inspired by her IVF journey, which was accompanied by her own personal photography archive, documenting medication and passing time. Engaging with archival material helped us reveal a continuum of embodied experiences that resist easy categorization and inspire creative work.

We displayed doctoral researchers in English and creative writing, who created artworks that directly responded to their research and foregrounded how societal and structural conditions shape experiences of health, identity, and access. Megan Constable’s doctoral project “The Right to Write,” includes a full-length Young Adult novel featuring a blind protagonist: *Autumn’s Ambition *(Fig. 6). She created a birthday card artwork in Braille formed by adhesive gems which matches the one Autumn received from an admirer. She also recorded screen read extracts from the novel, which were made available to visitors via QR codes, in order for them to hear how a visually impaired person might access the novel. Rai Powell created an installation from objects included in from her forthcoming young adult novel *After the Blood* about an autistic young woman experiencing menarche (Fig. 7). Installation provided a way to visualize the creative writing process, allowing for a unique way to engage with work-in-progress of creative writing and the objects that inform and enrich narrative.

**Fig. 6:**
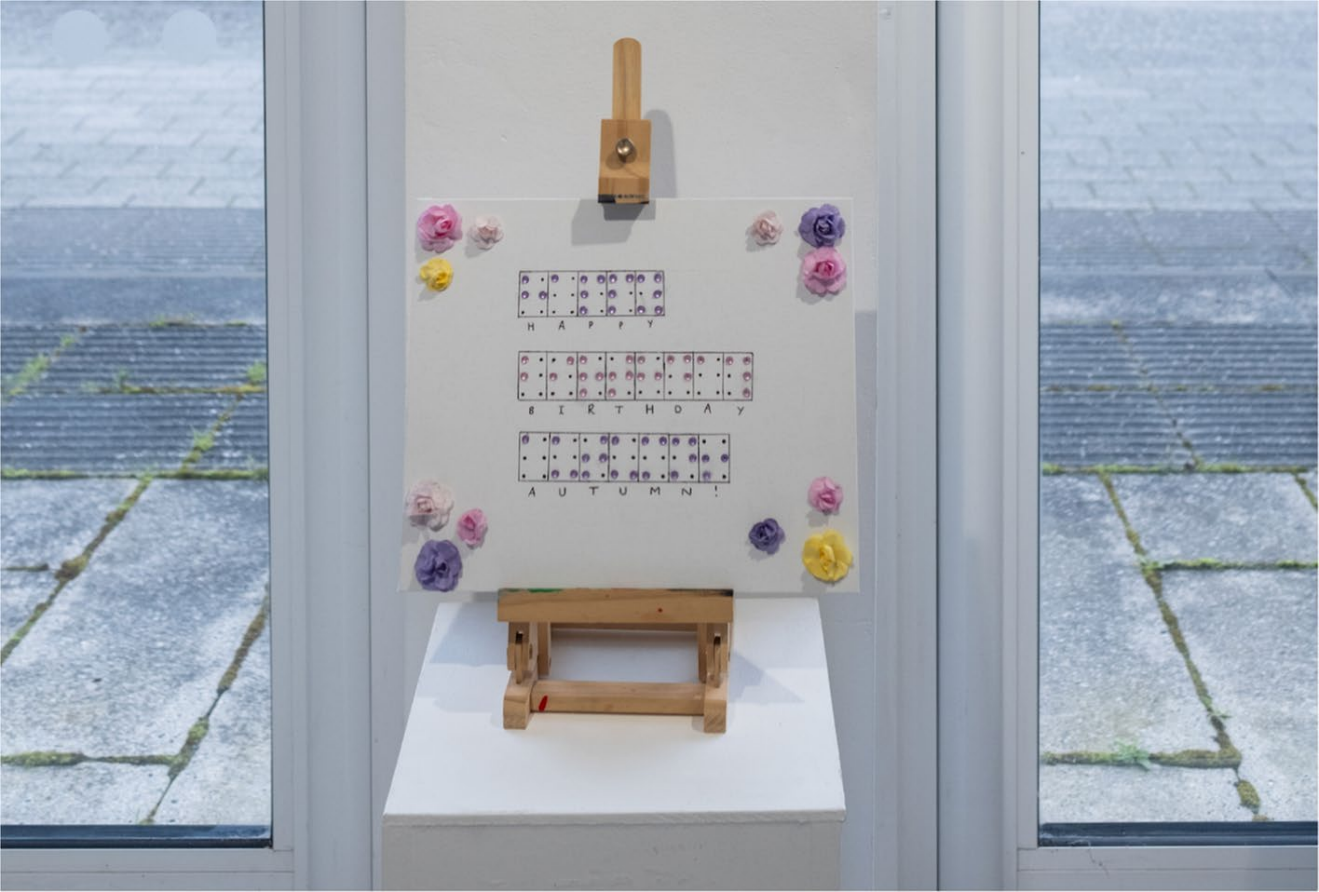
Megan Constable’s crafted Braille, representing *The Right to Write.* Image credit LU Arts, Loughborough University

**Fig. 7:**
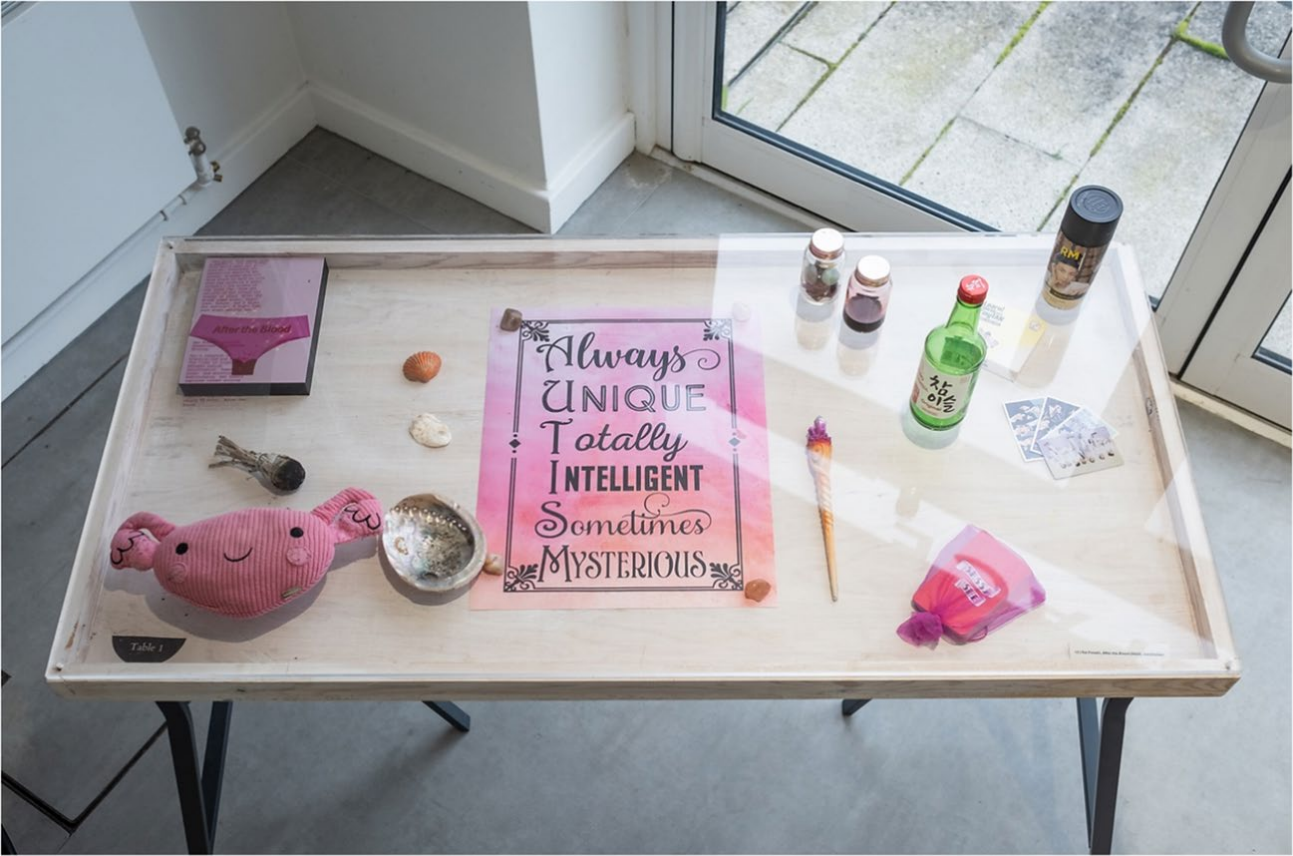
Dr Rai Powell’s installation, representing significant objects mentioned in her forthcoming young adult novel *After the Blood.* Image credit LU Arts, Loughborough University

The month-long exhibition showcased the depth and range of lively objects with which health humanities researchers engage to enhance and communicate their work. We hoped to demonstrate how ill-health and wellness is not a binary but rather a complex proposition, which alters in the context of where a person is on their journey through the life course. Overall, our curatorial rationale was to showcase how narrative and storytelling, and the methods of literary criticism, can be brought to life through objects, archival documents, and creative responses. In the exhibition, notecards offered a poignant tool to cope with loss, archival records were turned into found poems, and stitches and breath became new types of life writing. By foregrounding interpretive practices, we suggest that literary scholars can construct and reveal intertextual, affective, and often speculative connections across time in relation to health, illness, and wellbeing.

## Endnotes

^1^ Including early modern women’s health, pregnancy, and childbirth; early modern dietary culture; nineteenth-century mental health; and representations of aging, care, and labor the twentieth and twenty-first century. Find more information at https://www.lboro.ac.uk/subjects/english/research/groups/health-humanities/.

## Data Availability

The data that support the findings of this study are openly available in the Loughborough University repository at: https://repository.lboro.ac.uk/articles/event/Health_From_Cradle_to_Grave_Birthing_Chair_to_the_Death_Couch/25783704.

## References

[CR1] Barratt, Harriet. 2018. Revise your orthodoxies: A response to “Curating the medical humanities.” *The Polyphony*. https://thepolyphony.org/2018/10/17/revise-your-orthodoxies-a-response-to-the-curating-the-medical-humanities-workshopbirkbeck-13th-september-2018/. Accessed 1 March 2025.

[CR2] Joshi, Swati. 2022. ‘Documents of my breath’. *Wellcome Collection: Stories*. https://wellcomecollection.org/stories/documents-of-mybreath. Accessed 1 March 2025.

[CR3] Muller, Lizzie, and Caroline Seck Langill, eds. 2021. *Curating lively objects: Exhibitions beyond disciplines.* London: Routledge.

[CR4] Shakespeare, Tom. 2010. The social model of disability. In *The disability studies reader,* ed. by Lennard J. Davis, 266-73. New York: Routledge.

